# Modelling Organic Gel Growth in Three Dimensions: Textural and Fractal Properties of Resorcinol–Formaldehyde Gels

**DOI:** 10.3390/gels6030023

**Published:** 2020-08-05

**Authors:** Elisha Martin, Martin Prostredny, Ashleigh Fletcher, Paul Mulheran

**Affiliations:** Department of Chemical and Process Engineering, University of Strathclyde, Glasgow G1 1XJ, UK; elisha.martin@strath.ac.uk (E.M.); martin.prostredny@strath.ac.uk (M.P.); ashleigh.fletcher@strath.ac.uk (A.F.)

**Keywords:** gel modelling, RF gels, nanomaterials, cluster aggregation, gel formation, fractal analysis, aerogels, xerogels

## Abstract

Tailoring the properties of porous organic materials, such as resorcinol–formaldehyde gels, for use in various applications has been a central focus for many studies in recent years. In order to achieve effective optimisation for each application, this work aims to assess the impact of the various synthesis parameters on the final textural properties of the gel. Here, the formation of porous organic gels is modelled using a three-dimensional lattice-based Monte Carlo simulation. We model growth from monomer species into the interconnected primary clusters of a gel, and account for varying catalyst concentration and solids content, two parameters proven to control gel properties in experimental work. In addition to analysing the textural properties of the simulated materials, we also explore their fractal properties through correlation dimension and Hurst exponent calculations. The correlation dimension shows that while fractal properties are not typically observed in scattering experiments, they are possible to achieve with sufficiently low solids content and catalyst concentration. Furthermore, fractal properties are also apparent from the analysis of the diffusion path of guest species through the gel’s porous network. This model, therefore, provides insight into how porous organic gels can be manufactured with their textural and fractal properties computationally tailored according to the intended application.

## 1. Introduction

The application potential for porous organic materials has been investigated extensively over the years, with a particular focus on those which possess attractive properties, such as low densities and high surface areas. Materials such as these have proven to be effective in a wide range of applications, many of which are imperative in reducing or eradicating detrimental environmental impacts of industry, heightening their pertinence to recent research. To date, applications for porous organic materials have included gas adsorption and storage [1,2], water treatment [3,4], and thermal insulation [5,6], as well as use in their carbonised forms for applications involving electrical conductivity [4,7].

This work focuses on one such class of organic porous materials—resorcinol–formaldehyde (RF) gels—which are formed via a sol–gel process and subsequently dried, producing the lightweight, nanoporous structure of the final gel. Despite extensive research into these porous materials in recent years, their formation mechanism is not yet fully understood, and their application potential is yet to be fully elucidated. Understanding the mechanism by which these materials form is crucial in determining how various synthesis parameters affect the final structural properties of the gel, and modelling this computationally could permit future optimisation of materials according to their relevant application.

The synthesis of these gels involves a base-catalysed addition reaction between resorcinol and formaldehyde molecules, resulting in the formation of hydroxymethyl derivative monomers. A condensation reaction proceeds as these monomers become interconnected via methylene and methyl-ether bridges, with the resulting compounds forming primary spherical clusters. The condensation reaction continues, with the eventual aggregation of these primary clusters leading to the cross-linked network structure of the final gel [8]. 

**Figure 1:**
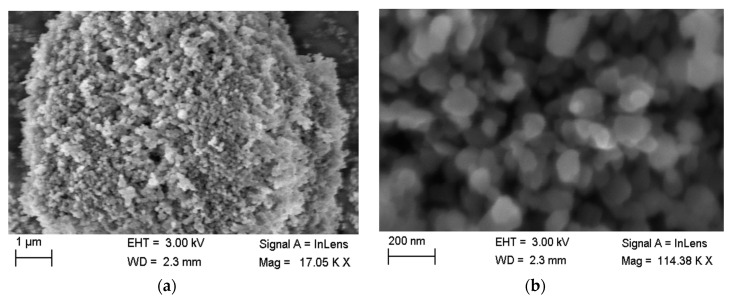
SEM images of resorcinol–formaldehyde (RF) xerogels synthesised at 20% solids content and a resorcinol/catalyst ratio of 600 at (**a**) 17.05 K X magnification and (**b**) 114.38 K X magnification. This SEM analysis was performed in collaboration with Farnaz Ghajeri at Angstrom Laboratory, Uppsala University.

The proposed mechanism by which this growth process is initiated begins with the abstraction of a proton from resorcinol in the presence of the basic catalyst, resulting in increased reactivity of the anionic resorcinol molecule [9]. This subsequently acts as a cluster seed around which other monomers can attach, forming the primary spherical particles. The resulting structure can be observed experimentally using Scanning Electron Microscopy (see Figure 1), as has been reported in previous studies on RF gels [10,11].

This growth pathway forms the basis for the computational work presented here, a three-dimensional (3D) simulation that models the formation and growth of porous materials, such as RF gels, using a kinetic Monte Carlo methodology. This software has been developed in-house and builds upon a previous 2D simulation from within our group [12]. 

Substantial computational research into basic cluster–cluster aggregation systems has been carried out over the years, which have simulated the formation of complex structures from both diffusion-limited and reaction-limited cluster aggregation [13,14,15]. Recent studies have furthered this work, with a focus on the fractal properties of systems modelled with repulsive and attractive forces in place, and the rotational diffusion of aggregating clusters implemented [16,17]. The model presented here uses a novel approach to cluster–cluster aggregation to simulate the formation of porous materials, originating from the initial monomer species. The subsequent growth of primary clusters around cluster seeds, therefore, allows for primary clusters of varying sizes to form, before their final aggregation into monolithic, porous structures. This is in contrast to previous studies, which have focused on the cluster aggregation process for similar porous materials, many beginning the simulation at a point where primary cluster formation had already taken place [18,19], or assuming primary clusters which have formed are of equal size before aggregation occurs [20,21]. These models, therefore, are not reflective of a real system, where such properties are likely to exhibit some degree of variation. Although the model presented in this work does not account for additional forces or rotational effects exhibited by real systems, basic diffusional moves sufficiently capture the stochastic nature of the growth given that the aggregation process is modelled within a crowded environment.

Various analytical techniques have been applied to gain a deeper insight into the internal structural properties of porous materials, using both experimental and computational methods. Experimentally, adsorption analysis in particular has been used to provide details on the accessible pore volume of RF gel materials, in addition to their accessible surface area, average pore width, and approximate pore geometry [22,23]. In addition to this, assessing whether these materials have fractal properties has also been addressed, since the scaling properties of a porous material need to be properly recognised when designing it for an application. 

Over the years, research has been conducted into the fractal properties of RF gels using Small-Angle X-Ray Scattering (SAXS) measurements, the results of which are used to determine surface fractal dimension values, with values below three indicating fractal properties. The studies carried out to date have reached conflicting conclusions, with some categorically concluding that RF gels—unlike their silica gel counterparts—do not possess any fractal properties whatsoever. This includes a study by Pekala (who was the first to synthesise RF gels in 1989 [24]) and Schaefer (1993) [25] who conducted SAXS analysis on base-catalysed RF aerogels. The results obtained show no fractal properties for any of the materials studied, although the authors suggest that fractal behaviour may be possible for samples synthesised with particularly low densities, which is something explored in this work. The results of subsequent studies have pointed towards the possibility of some fractal properties of the gels synthesised under certain conditions, with a general consensus yet to be reached. 

Tamon and Ishizaka (1998) [26] assessed the fractal properties of RF gels at different time intervals throughout their gelation process, as well as after a period of ageing. While they find evidence of fractal structures during growth, the final gel structures did not display fractal properties in SAXS measurements. Berthon et al. (2001) [27] also carried out SAXS analysis of RF gels which had been synthesised at solids percentages of 5% and 20% using both acidic and basic reaction conditions, as well as using both acetone and water as solvents for the sol–gel process. The results of this work indicated that fractal properties could be observed for RF gels synthesised at low solids percentages (5%) under acidic reaction conditions using acetone as a solvent, with a calculated surface fractal dimension value of 2.5. RF gels synthesised at higher solids percentages (20%), however, did not exhibit any fractal properties. More recently, research published by Alshrah et al. (2018) [28] analysed the relationship between fractal and thermal properties within these materials. Gels were synthesised at different catalyst concentrations and solids percentages, both of which remained low across the ranges studied, and surface fractal dimension values were determined following SAXS analysis, with values pointing towards fractal properties. 

The dichotomous conclusions reached as a result of the different studies carried out over the years reinforce the unanswered questions around the fractal properties of RF gels. Furthermore, in our group’s earlier two-dimensional model, which simulated the formation of RF gels, the resulting structures did exhibit fractal properties, even at the higher solids percentages studied. Of course, two-dimensional systems will have more restricted percolation pathways than the three-dimensional structures observed in reality, consequently influencing their fractal properties. The work presented here, therefore, aims to explore this further, this time using a three-dimensional computational model to determine fractal dimension values of the simulated material at various solids percentages and catalyst concentrations. Reflective of the materials studied experimentally by SAXS analysis in the different studies discussed, one would anticipate that the simulated structures may exhibit some fractal properties at sufficiently low solids percentages, while appearing largely nonfractal at the higher solid percentages, which are more commonly used for material synthesis in experimental analysis. 

The various porous structures produced through the 3D simulation developed in this work are analysed in terms of their textural and fractal properties, and compared not only to one another, but also to materials that have been examined experimentally. The transformation of this model from 2D to 3D has been crucial in achieving an accurate comparison to materials synthesised through experimental work, and is a pivotal step towards achieving computational optimisation of these materials for use in various applications.

## 2. Results and Discussion

### 2.1. Visualisation and Cluster Size

**Figure 2:**
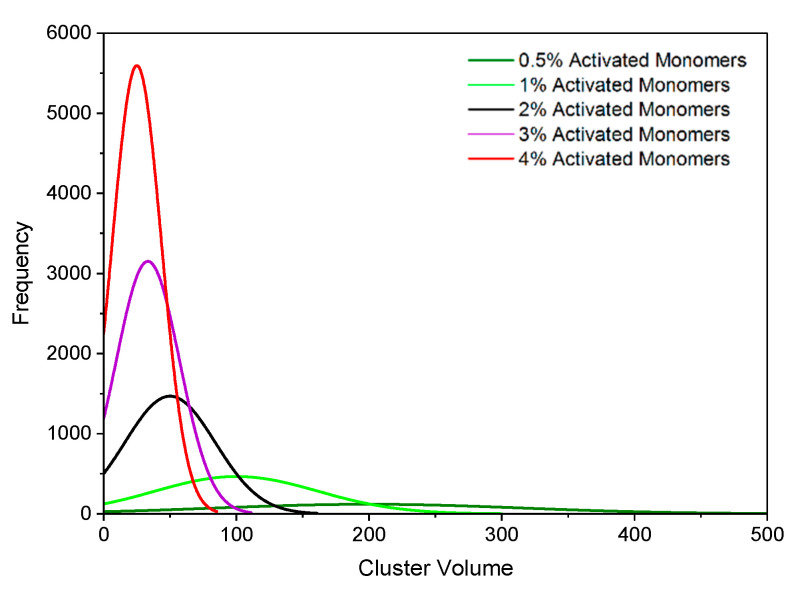
Histogram plots of primary cluster volume distributions at activated monomer ($C_{c}$) values of 0.5%–4%. Data are presented for 60% solids content ($S_{c}$).

**Figure 3:**
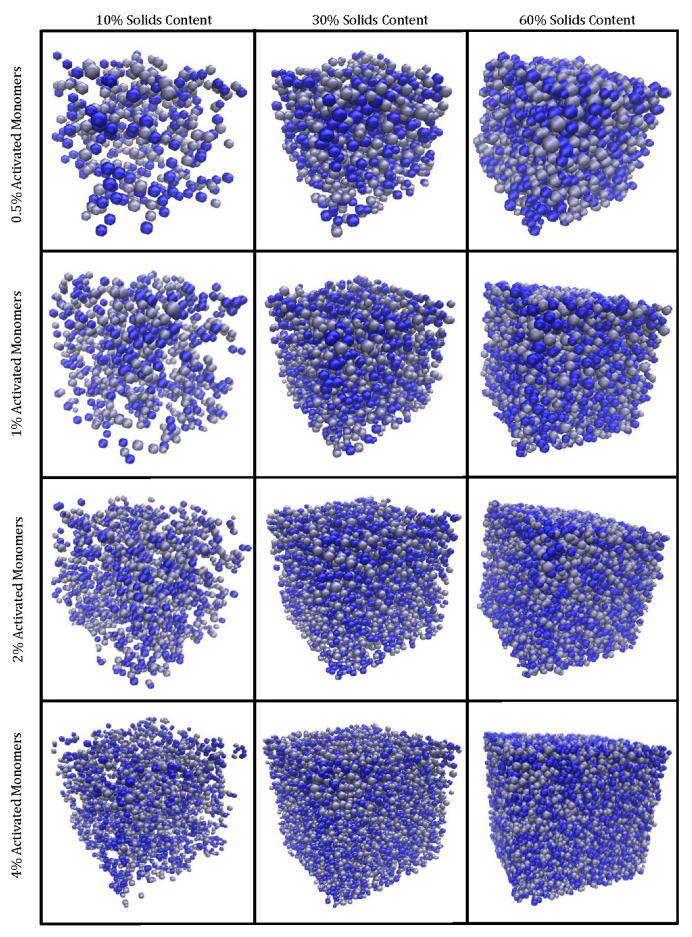
Simulated RF gel materials visualised in 3D, with increasing solids content ($S_{c}$), left to right, and an increasing percentage of activated monomers ($C_{c}$), top to bottom. Note that each sphere represents an individual cluster, and the different colours of clusters present are for visual purposes only.

Figure 2 displays histograms for the primary cluster volume distributions at various catalyst concentrations, while Figure 3 shows the visualised final structures. As explained in the Methodology section, $S_{c}$ is the solids content in the simulation and $C_{c}$ is the catalyst concentration, mirroring experimental conditions. The simulation yields approximately spherical “primary particles” that have also aggregated to form the gel structure. Figure 3 displays structures created with $S_{c}$ values of 10%, 30%, and 60% each at $C_{c}$ values of 0.5%, 1%, 2%, and 4% on a 100 × 100 × 100 site lattice. The structures visualised are monolithic, despite some clusters at the edges appearing unattached; these are connected to the structure via periodic boundaries. GIFs of the simulated materials can also be found in the Electronic Supporting Information (available from the University of Strathclyde KnowledgeBase [29]).

The visual differences between the structures at various $S_{c}$ values are evident. Higher $S_{c}$ results in materials that, as expected, are more densely packed, with the primary clusters occupying more space within the lattice. When the average primary cluster sizes within the structures are compared, materials with the same $C_{c}$ possess the same average volume and radius regardless of $S_{c}$. A structure simulated at a higher $S_{c}$ will, however, have a greater number of primary clusters within its lattice in comparison to one at lower $S_{c}$ at the same $C_{c}$ value. This means that, although an increase in $S_{c}$ results in an increase in monomers within the lattice, these monomers are distributed across a greater number of primary clusters, therefore, resulting in the average primary cluster size remaining constant across the different $S_{c}$ values. 

On the other hand, $C_{c}$ has a significant impact on the average primary cluster size within the structure, as shown in Figure 2. As $C_{c}$ increases from 0.5% to 4%, the average primary cluster volume decreases from 200 to 25 lattice sites. This is consistent with observations from experimental analysis of RF gels; materials synthesised with low catalyst concentrations comprise of fewer primary clusters that are larger in size, while those synthesised with high catalyst concentrations comprise of a greater number of primary clusters that are smaller in size [8]. 

### 2.2. Accessibility of Sites

**Figure 4:**
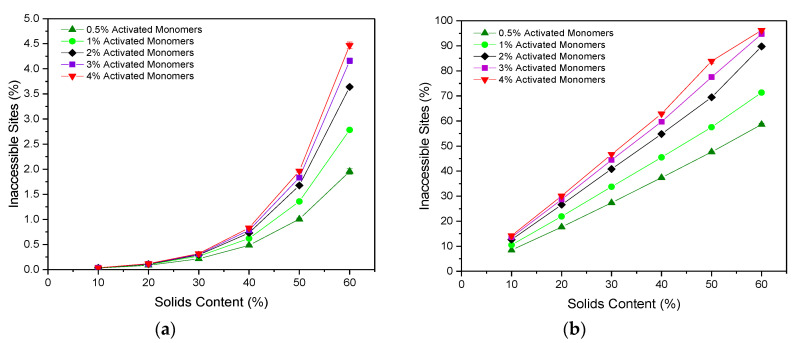
Percentage of inaccessible sites within the lattice with varying solids content ($S_{c}$) and activated monomer ($C_{c}$) values with respect to a diffusing particle of (**a**) Size 1 and (**b**) Size 3. Standard deviation error bars are present around each data point, although may not be visible due to their size relative to the data point marker.

The accessibility of pore sites within a porous material is a fundamental consideration when it comes to their application potential, and is, therefore, an important property to analyse within simulated structures. We consider how the accessibility is affected by the size of the guest species, to understand how this might affect potential applications of the porous gel as a host for different molecules. As expected, the percentage of sites that are inaccessible increases with increasing $S_{c}$, as the lattices are more densely packed with material and, therefore, more likely to result in closed-off porosity. This is true for the accessibility of particles both of one and three sites in size (corresponding to molecular size of approximately 1 and 3 nm), as shown in Figure 4a,b, respectively. Furthermore, the percentage of inaccessible sites also increases with increasing $C_{c}$, which is a result of the increased number of clusters present. Structures formed at higher $C_{c}$ possess a greater number of initial cluster seeds than those at lower values, leading to the formation of numerous smaller clusters, which pack together densely, increasing the likelihood of closed-off porosity.

The percentage of sites inaccessible to a particle of Size 1 remains consistently low, ranging from 0.028(2)% at 10% $S_{c}$ and 0.5% $C_{c}$ to 4.47(7)% at 60% $S_{c}$ and 4% $C_{c}$. These values are considerably lower than those obtained for the 2D version of the code, which reached up to 25% inaccessible sites for structures formed with 50% $S_{c}$ and 3% $C_{c}$ [12]. Simulating the porous structure in three dimensions opens new accessible pathways for connectivity, which would otherwise be limited by the two-dimensional structure, explaining the significant decrease in the percentage of inaccessible sites within the lattice, and providing a more accurate representation of the porous materials synthesised in reality.

When the particle size is increased from one site to three sites, the percentage of inaccessible sites increases significantly across all $S_{c}$ and $C_{c}$ values, and the values obtained span a much wider range. In this case, values range from 8.45(3)% at 10% $S_{c}$ and 0.5% $C_{c}$ to 96.24(8)% at 60% $S_{c}$ and 4% $C_{c}$. These results have significant implications for porous materials in their potential use for applications involving larger particles such as biomolecules, where optimisation of $S_{c}$ and $C_{c}$ values according to particle size would be imperative, ensuring that the synthesis parameters used produce structures with sufficiently accessible porous networks.

### 2.3. Surface Area

**Figure 5:**
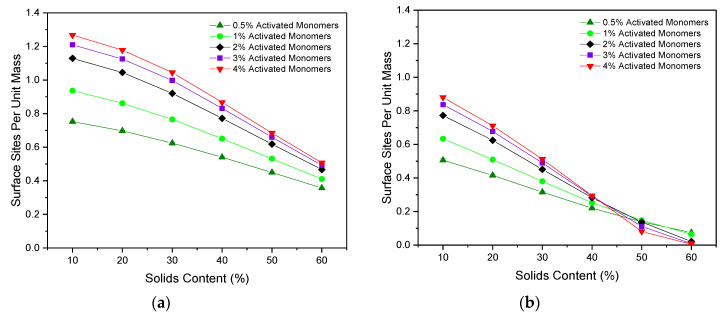
Accessible surface sites scaled with total mass with varying solids content ($S_{c}$) and activated monomer ($C_{c}$) values with respect to a diffusing particle of (**a**) Size 1 and (**b**) Size 3. Standard deviation error bars are present around each data point, although may not be visible due to their size relative to the data point marker.

Figure 5a,b shows the accessible surface area per unit mass for particles of Sizes 1 and 3, respectively. For both particle sizes, the accessible surface area per mass gradually decreased as $S_{c}$ increased across each of the $C_{c}$ values studied. The increased number of primary clusters present for higher $S_{c}$ (at a given $C_{c}$) results in structures that are more densely packed, as previously discussed. This increases the likelihood that a single primary cluster will be in contact with multiple primary clusters around it, therefore reducing the accessible surface area available for particles moving through the porous structure. Furthermore, as expected, the accessible surface area is consistently higher for a particle of Size 1 than for Size 3, as the smaller particle can more easily access the narrower pores within the structure.

The effect of variations in $C_{c}$ can also be compared, the results of which indicate that, for a particle of one site in size, an increase in $C_{c}$ value leads to an increased accessible surface area across the $S_{c}$ values studied. As previously discussed, higher $C_{c}$ values lead to a greater number of primary clusters present, across which the structure’s mass is distributed. Consequently, for lower $C_{c}$ materials, the larger primary clusters mean that much of the structure’s mass is contained within the interior of each cluster, reducing the accessible area available at the surface. Conversely, for higher $C_{c}$ materials with a greater number of primary clusters present, each of which is smaller in size, the accessible area available at the surface is increased. Similar trends for accessible surface area were also observed for the 2D version of this simulation for a particle of one site in size, although the work presented here explores a wider range of $S_{c}$ and $C_{c}$ values, as well as including the new analysis for a particle of three sites in size.

Importantly, these results are also consistent with those observed experimentally, where an increase in catalyst concentration is shown to increase the BET surface area values obtained from nitrogen adsorption measurements of RF gels [25,30].

When the particle size is increased to three sites, the same initial trend is observed where an increase in $C_{c}$ leads to higher values of accessible surface area, however, an eventual crossover point is reached at an $S_{c}$ value of around 45%. For $S_{c}$ values above this point, increasing $C_{c}$ has the inverse effect, where the accessible surface area is hindered by higher $C_{c}$ values. This likely arises due to the high percentage of inaccessible sites for particles of Size 3, which is exacerbated by the increased interconnectivity arising at higher $C_{c}$ values. An upper limit is therefore reached, where the increased interconnectivity associated with the greater number of primary clusters present is no longer of benefit to the available surface area of the system. Instead, it gives rise to higher rates of closed-off porosity and therefore reduces the accessibility of surface sites—an important consideration for the tailoring of these materials to various applications.

### 2.4. Correlation Dimension

The correlation dimension ($D_{c}$) of a structure is a measure of its fractal properties, with uniformly distributed, densely packed structures in three dimensions having $D_{c}=3$, and fractal structures conversely having $D_{c}<3$. As previously discussed, questions around the fractal nature of RF gels have been raised over the years with a consensus yet to be reached, therefore calculating $D_{c}$ for the simulated structures could be pivotal in addressing some of the unanswered questions.

**Figure 6:**
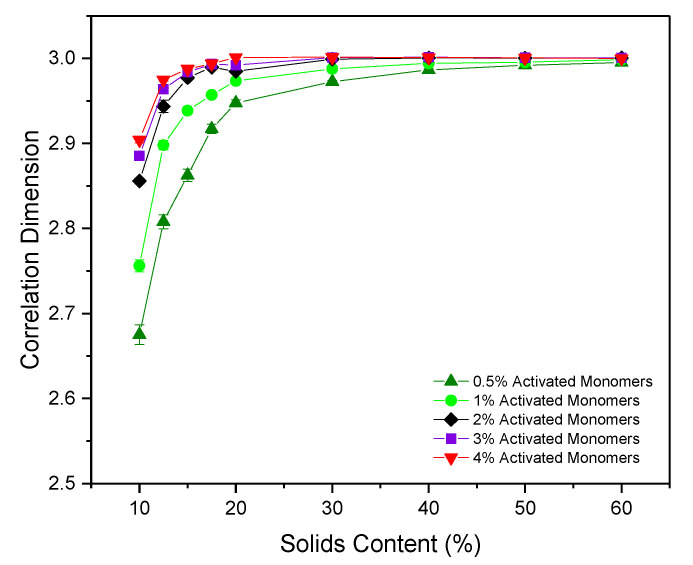
Correlation dimension values calculated for simulated materials at varying solids content ($S_{c}$) and activated monomer ($C_{c}$) values. Standard deviation error bars are present around each data point, although may not be visible due to their size relative to the data point marker.

Figure 6 shows $D_{c}$ for the simulated structures at various $S_{c}$ and $C_{c}$, with most data provided between 10% and 20% $S_{c}$ where the most significant changes in $D_{c}$ are observed. For each $C_{c}$, a gradual increase in correlation dimension is observed as $S_{c}$ increases from 10% to 20%, shortly thereafter plateauing around a value of 3, the value at which a structure is considered to possess no fractal properties. At the lowest $S_{c}$ of 10%, the structure possesses $D_{c}$ values of 2.68(1) and 2.76(1) for $C_{c}$ values of 0.5% and 1%, respectively, indicating that the structures do exhibit some fractal properties under these conditions. These lower limit values are approaching that which would be expected of dilute cluster aggregation systems, determined to possess $D_{c}$ values of ~2.5 [31]. For higher $C_{c}$ structures at 10% $S_{c}$, the $D_{c}$ value approaches 2.9, close to the nonfractal limit of three. These results indicate that fractal properties can be observed within these materials under specific synthesis conditions—reliant not only on sufficiently low $S_{c}$, as previously postulated, but also on sufficiently low $C_{c}$ values. Under standard gel synthesis conditions within experiments, $S_{c}$ values of 20% and above are commonly used, perhaps explaining why numerous studies have observed no fractal properties within the structures.

These results differ from those obtained from the 2D simulation, where the value of a uniformly distributed, densely packed structure yields $D_{c}=2$, with fractal structures having $1<D_{c}<2$. For the 2D model, $D_{c}$ values obtained ranged from as low as ~1.55, and gradually increased with increasing $S_{c}$ and $C_{c}$. $D_{c}\;$ slowly reached a plateau at a value of two between 40% and 50% $S_{c}$, in contrast to the faster convergence of values within the 3D analysis. As previously discussed, 2D systems will have more restricted percolation pathways than in 3D structures, consequently influencing their fractal properties, explaining the disparity in calculated values across the two models. The work from the 2D model consequently concluded that the materials did, in fact, possess fractal properties, even those which had been synthesised at higher $S_{c}$ and $C_{c}$. In light of the results presented here, this conclusion should now be revised.

### 2.5. Hurst Exponent for Diffusion through the Porous Structures

**Figure 7:**
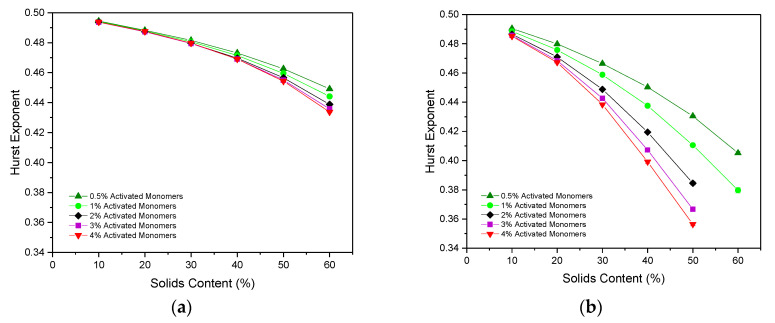
Hurst exponent values calculated for varying solids content ($S_{c}$) and activated monomer ($C_{c}$) values with respect to a diffusing particle of (**a**) Size 1 and (**b**) Size 3. Standard deviation error bars are present around each data point, although may not be visible due to their size relative to the data point marker. Note that a sufficiently percolated structure could not be identified for structures above 1% $C_{c}$ at 60% $S_{c}$ for a particle of three sites in size, hence the missing values.

As discussed in the Methodology section, the Hurst exponent (H) of a particle moving through an empty lattice should be 0.5, indicating regular Brownian motion, with values below this pointing towards antipersistent motion. Figure 7a,b displays the H values calculated for simulated structures with various $S_{c}$ and $C_{c}$ values, for particles of Sizes 1 and 3, respectively. In both cases, the H value decreases with increasing $S_{c}$, as the path of the random walker becomes more obstructed due to the increased number of occupied sites densely packed within the lattice, directly affecting the particle’s motion through the porous network.

The value of H also decreases as the value of $C_{c}$ increases, this time as a result of the increasingly complex, interconnected structures formed from the greater number of primary clusters present. These complex structures create additional obstructions within the path of the random walker, hindering its ability to diffuse freely throughout the lattice and, therefore, decreasing the value of H.

The H values obtained for a random walker of Size 1 range from 0.4945(2) at 10% $S_{c}$ and 0.5% $C_{c}$ to 0.4338(2) at 60% $S_{c}$ and 4% $C_{c}$. As these values are all below 0.5, they indicate that the random walker motion is antipersistent in nature, as previously discussed. For materials simulated at low $S_{c}$ and $C_{c}$, this value falls only slightly below 0.5 due to the largely open, sparsely-populated structure within the lattice. These H values differ slightly from those cited for the 2D model, where the lowest value obtained reaches below ~0.36 for 50% $S_{c}$ and 3% $C_{c}$. Similar to the comparative analysis of inaccessible sites between the 2D and 3D models, this disparity in H values arises as a result of the new pathway for accessibility opened by the 3D simulation. Once again, opening the structure to the third dimension allows the random walker to diffuse around the lattice more freely, and more accurately reflects how a particle might diffuse through a porous material in reality.

When the random walker size is increased from one to three, the H value obtained decreases far more rapidly as its ability to move around the lattice is restricted by its width. In this case, H values range from 0.4904(2) at 10% $S_{c}$ and 0.5% $C_{c}$ to 0.356(1) at 50% $S_{c}$ and 4% $C_{c}$, close to the limit of 1/3 that is expected at the percolation threshold [12]. As with the analysis of the inaccessible sites for a particle of three sites in size, assessing the motion of such a particle through a porous material in this manner provides valuable insight for their use in applications involving diffusion of larger particles. Note that a sufficiently percolated pore structure could not be identified for materials formed using 60% $S_{c}$ at $C_{c}$ values above 1% for a particle of Size 3, meaning that the porosity was too closed-off for the particle to freely diffuse through the structure.

**Figure 8:**
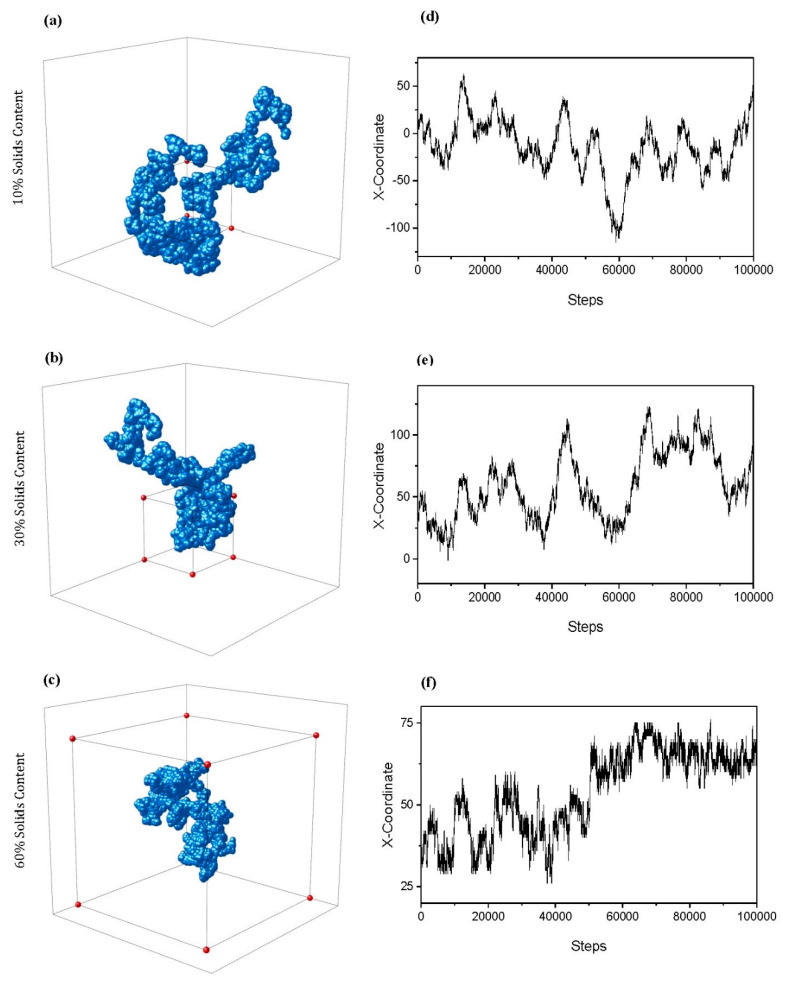
Example 3D traces for particles of three sites in size diffusing through simulated structures with 1% activated monomers ($C_{c}$) and solids content ($S_{c}$) values of (**a**) 10%, (**b**) 30%, and (**c**) 60%. Note that the axes of each trace differ dependent on the extent to which the particle was able to diffuse through the lattice across periodic boundaries—a box of size 100 × 100 × 100 sites is included within each trace to allow a comparison of scale. Corresponding x coordinate traces for each structure at $S_{c}$ values of (**d**) 10%, (**e**) 30%, and (**f**) 60%.

The calculation of H also enables a 3D visual trace of how a particle might move through the porous structure, as shown in Figure 8a–c, for $S_{c}$ values of 10, 30, and 60%, respectively. All visualised traces are for particles of Size 3 and $C_{c}=1\%$. Note that the axes of each trace differ depending on the extent to which the particle was able to diffuse through the lattice across periodic boundaries—a box of 100 × 100 × 100 sites in size has been included to allow a comparison of scale. As $S_{c}$ increases, the path of the random walker becomes more obstructed, increasing the likelihood that it will turn back on itself as it diffuses around the lattice. This is reflected in the 3D traces visualised, where the particle is unable to explore the lattice to the same extent in the 60% $S_{c}$ structure in comparison to the 10 or 30% $S_{c}$ structures within the same number of steps. The periodic boundaries in place allowed the particle to continue to explore the lattice out with the 100 × 100 × 100 original size, demonstrated particularly within the 10% $S_{c}$ trace (Figure 8a) in addition to that of 30% (Figure 8b). This is in contrast to the 60% $S_{c}$ diffusing particle (Figure 8c), whose path was far more limited in terms of the extent to which was able to explore the densely packed lattice. Figure 8d–f displays the traces of the x coordinate of the particle as it diffuses through the respective lattice, further highlighting the changing diffusive behaviour as the porous networks become more complex and constricted with increased $S_{c}$. These traces provide further insight into the internal percolated structure of the simulated material, and demonstrate the antipersistent nature of the particle’s motion as it diffuses.

## 3. Conclusions

The formation mechanism of porous materials such as resorcinol–formaldehyde gels is captured in this work through the development of a 3D cluster growth and aggregation model. The model explores the effect of activated monomer percentage—a parameter that mimics catalyst concentration—and solids content, and allows comparisons to be drawn between the simulated materials and those synthesised in the lab. The resulting simulated material is a monolithic structure of interconnected primary (approximately spherical) clusters, consistent with structures observed experimentally.

Structural analysis of the simulated material was carried out across each solids content and activated monomer percentage studied, including pore accessibility and available surface area. Materials simulated with higher solids contents exhibited a higher percentage of inaccessible pore sites and reduced accessible surface area, both of which are as a result of the densely packed structures. Materials simulated with higher activated monomer percentages, on the other hand, were composed of a greater number of primary clusters that were smaller in size, leading to structures that exhibited an increase in accessible surface area for the diffusion of a particle of Size 1. For a particle of Size 3, this increase in accessible surface area was observed until an upper limit at a solids content of ~45%, after which the increased interconnectivity was no longer of benefit to the available surface area of the system. Instead, it gave rise to higher proportions of closed-off porosity, consequently reducing the accessibility of surface sites within the structure.

An important aspect of this research was to further explore the fractal properties of RF gels under varying synthesis conditions, and so the correlation dimensions of the simulated structures were calculated. The results obtained indicate that fractal properties can be observed within RF gel materials under specific synthesis conditions—reliant not only on sufficiently low solids content, as previously postulated, but also on sufficiently low catalyst concentrations. Under standard gel synthesis conditions within experiments, solids contents of 20% and above are commonly used, perhaps explaining why numerous studies have previously observed no fractal properties within the structures. This analysis sheds some light on the ongoing debate over the fractal properties of RF gels, and could explain the conflicting conclusions drawn from different experimental studies.

Hurst exponents for particles diffusing through the material’s porous network were also calculated, the results of which point towards the antipersistent motion of the particle. The degree of antipersistence was exacerbated by increasing solids and catalyst concentration, as well as the increase in width from a diffusing particle of one site to three sites in size. Analysing the way in which a particle of varying size diffuses through these porous materials is an important consideration for their application potential and subsequent optimisation, particularly for applications involving larger particles such as enzymes and antibodies. Furthermore, while the correlation dimension of the material (as measured through SAXS, for example) might not reveal fractal properties, the application of the material as an absorbent still requires consideration of the fractal nature of material diffusion through the porous structure.

This 3D simulation is a continuation of work from a 2D model, which operates under the same principles, with the progression to three dimensions providing a more accurate representation of the materials synthesised in reality. A direct comparison between specific experimental and computational materials is complex, however, due to the intricate nature of the resorcinol–formaldehyde reaction, in addition to the various synthesis conditions that significantly impact gel properties. As discussed, the range of values used for activated monomer percentages is based around the percentage of resorcinol molecules expected to be deprotonated in the presence of a basic catalyst, and, although the model successfully captures the trends in material properties as catalyst concentration is altered, an exact comparative experimental resorcinol/catalyst (R/C) ratio has not yet been established. Future work that uses sorption analysis to compare experimental and computational isotherms, allowing a more direct comparison to be drawn between activated monomer percentages and specific R/C ratios, is planned within our research group. Furthermore, the adaptation of this model to reflect acid-catalysed gels, where the final material comprises branched chains of spherical particles as opposed to aggregated clusters, could be of interest.

## 4. Materials and Methods

### 4.1. Simulation Procedure

The 3D model presented here builds on the work published by Prostredny et al. [12]—a 2D simulation which operates under the same principles—where a detailed description of the simulation process can be found. Here, a cubic lattice of Size 100 × 100 × 100 sites, totalling 1,000,000 sites, was initially populated at random with monomers according to the desired percentage solids content ($S_{c}$), an important parameter in the synthesis of RF gels in laboratory experiments. In this research, solids contents of 10%–60% were simulated, with values above this range proving to result in densely packed structures with pores that are predominantly inaccessible. This model was developed with GNU Fortran compiler and GNU parallel tool [32].

As previously discussed, the laboratory synthesis of RF gels includes a reaction between resorcinol and formaldehyde molecules with the addition of a basic catalyst, the presence of which leads to the formation of negatively charged resorcinol ions. These anionic molecules subsequently act as cluster seeds around which monomers can attach, leading to the formation of primary (approximately spherical) clusters. This process is modelled in the simulation by “activating” at random a percentage of the monomers on the lattice, with each activated monomer acting as a primary cluster seed for the simulation, and where the varying percentage of activated monomers is comparable to varying catalyst concentration ($C_{c}$). In this research, activated monomer percentages of 0.5%–4% are simulated, a range based on the proposed percentage of resorcinol molecules that are deprotonated by a basic catalyst during the RF reaction [33].

The simulation begins with the random diffusion (nearest-neighbour hopping) of monomers on the lattice, with periodic boundary conditions, during which free monomers attach to activated monomers when they come into contact, forming larger primary clusters of monomers. These monomers attach in an approximately spherical sequence, as described in the work by Prostredny et al. [12], producing primary clusters that also diffuse on the lattice following the same basic scaling laws for diffusion. Two diffusing clusters irreversibly attach when they meet, retaining the primary clusters intact. The probability of cluster diffusion is inversely proportional to its mass, which takes into account the aggregation of primary particles to create irregular aggregates for diffusion. We only include simple nearest-neighbour hops in this model, since the growth occurs in a crowded environment so that effects of rotational diffusion or finite reactivity are expected to be minimal; further work on this aspect could be undertaken if required to explain experimental data.

The simulation proceeds until there are no free monomers present and the entire lattice comprises one monolithic, interconnected aggregate structure. The final structure is a porous network comprising primary clusters aggregated together, similar to that observed for RF gels as evident in Figure 1. Note, however, that the simulation could also be applicable to other porous materials whose formation mechanism operates under similar principles.

Each simulation was repeated with 10 different random number seeds, resulting in 10 different structures at each value of $S_{c}$ and $C_{c}$. An average was then calculated for each of the properties analysed across the 10 structures at each $S_{c}$ and $C_{c}$, as well as the corresponding standard deviation of the values calculated.

### 4.2. Visualisation

Visualisation of these three-dimensional structures is important not only for a comparison between the different simulated materials, but also for visual comparison with materials synthesised experimentally. The 3D structures from this simulation are visualised using Visual Molecular Dynamics (VMD) software, with each primary cluster represented by a sphere, the coordinates of which are taken from the lattice and inserted into a VMD-readable file format. The average number of monomers within each primary cluster was taken to be an equivalent spherical volume, and the average equivalent radius was subsequently determined and used to visualise the clusters as spheres. In this way, a structure that is easy to visualise, while reflecting the most important characteristics in terms of primary particles and the aggregated gel structure, is generated.

### 4.3. Textural Analysis

The various textural properties of these structures, at varying $S_{c}$ and $C_{c}$, are analysed and compared, including accessibility of pore sites for particles of both 1 and 3 lattice sites in size (hereafter referred to as Sizes 1 and 3). The length scale of the lattice is comparable to that of the RF dimer at ~1 nm [12], so a particle of Size 3 is comparable to a typical globular protein. The percentage of accessible pore sites for a particle of Size 1 is calculated by determining the percolated network of accessible sites within the structure, then expressing the total number of accessible sites within this network as a percentage of the total number of unoccupied sites within the lattice. To analyse the accessibility for a particle of Size 3, an exclusion zone of 1 site thickness is added to the simulated structures.

The accessible surface area of each structure with respect to its mass was also analysed for particles of Size 1 and 3. For a particle of Size 1, the total number of unoccupied sites adjacent to the surface of the cluster structure is divided by the total number of monomer sites within the structure. A similar procedure is carried out for a particle of Size 3, this time counting only unoccupied sites adjacent to the exclusion zone.

### 4.4. Fractal Analysis

As previously mentioned, computationally determining the fractal properties of RF gel materials, in particular, is of interest, especially in light of the conflicting conclusions that have been reached in various experimental studies. Suitable characterisation methods include the box counting dimension ($D_{b}$), the information dimension ($D_{i}$), and the correlation dimension ($D_{c}$). Of these, the most common dimension estimate used to characterise a fractal material is the correlation dimension, which is the more computationally efficient to determine of the three, and is based upon the proximity of points within the structure to one another within a spanning radius. Its calculation firstly begins with the determination of the correlation sum ($C_{r}$), as established by Grassberger [34] using Equation (1):

(1) $$C_{r}=\frac{1}{N\left(N-1\right)}\overset{N}{\underset{i=1}{\sum }}\overset{N}{\underset{j=1;j\neq 1}{\sum }}\theta \left(r-\left|X_{i}-X_{j}\right|\right)\;\;\;\;\;\;\;\;\;\;\;\;\;\;\;\;\;\;\;\;\;\;\;\;\;\;\;\;\;\;\;$$

Here, θ is the Heaviside function, r is the spanning radius, N is the total number of randomly selected reference points within the structure, and $X_{i}$ and $X_{j}$ are the coordinates of the two points whose proximity are being analysed within the system. The Heaviside function (θ) is equal to 1 when $\left(r-\left|X_{i}-X_{j}\right|\right)$ returns a positive value, indicating that the separation of points i and j is within the spanning radius. Conversely, when $\left(r-\left|X_{i}-X_{j}\right|\right)\;$ returns a negative value, θ is equal to 0. This calculation is carried out across increasing values of spanning radius until the entire structure has been encapsulated and $C_{r}$ consequently reaches a plateau.

The correlation sum relates to the spanning radius in the following manner:

(2) $$C_{r}\propto r^{D_{c}}\;\;$$

where the exponent $D_{c}$ is the correlation dimension. Obtaining the value of $D_{c}$, therefore, involves a logarithmic plot of the correlation sum vs. the spanning radius.

In this work, the correlation dimension for the simulated material is calculated from N= 100,000 different reference positions within the structure. Each position is selected at random, and the spanning radius between two reference positions is calculated for all periodic images, with the lowest value used to determine the correlation sum using Equation (1). A logarithmic plot of $C_{r}$ vs. r is produced in accordance with Equation (2), where the central area of the graph is a straight-line plot with no size limitations affecting the results, as described in the work by Prostredny et al. [12]. The value of $D_{c}$ is subsequently determined from the gradient of this linear section of the plot, eliminating the potential for finite size effects to impact the conclusions drawn around fractal properties. Example plots used to determine $D_{c}$ are shown in Figures S1 and S2 in the Supplementary Materials.

Analysing the fractional Brownian motion trajectory of a particle moving through a porous material is another valuable way in which its fractal characteristics can be determined. This is quantified using the original rescaled range method to calculate the Hurst exponent from the particle trace in the x, y and z directions when the particle takes a “random walk”—a series of random steps throughout the structure. Three classifications of fractal Brownian motion have been established: (1) Antipersistent motion, where the particle has a tendency to turn back on itself and revisit previous positions; (2) Neutrally persistent motion, also known as regular Brownian motion, which is observed when the particle is free to move around a lattice with no obstructions; (3) Persistent motion, where the particle has a tendency to progress its path in a particular direction. The value of the Hurst exponent from the random walk will determine which class of motion is observed, with values below 0.5 indicating antipersistence, values of 0.5 exactly indicating regular Brownian, and values above 0.5 indicating persistence.

The particle displacement from its origin over time is used to calculate the Hurst exponent, where the relationship between the average displacement (∆B) across the x, y, and z directions and the time window $T_{s}$ is as follows:

(3) $$\bar{\left|∆B\right|}\propto {\left(T_{s}\right)}^{H}\;$$

Here, the exponent H is the Hurst exponent, evaluated as the gradient of the logarithmic graph of ∆B vs. $T_{s}$ [35]. Example plots used to calculate H are shown in Figures S3 and S4.

In order to determine H, a random walker is allowed to diffuse through the accessible pore sites within the lattice and its path analysed in the x, y and z directions. The random walker takes 100,000 random steps in total from 100 different starting positions on the percolated structure (determined as above), and the average displacement (∆B) for each time window size ($T_{s}$) is calculated from the 100 traces. Note that the value of H calculated here is, therefore, that of the percolated porous network contained within the structure and not of the solid structure itself.

## Supplementary Materials

Supplementary materials can be found at https://www.mdpi.com/2310-2861/6/3/23/s1.

## References

[1] Das S., Heasman P., Ben T., Qiu S. (2017). Porous Organic Materials: Strategic Design and Structure–Function Correlation. Chem. Rev..

[2] Xie L.-H., Suh M.P. (2013). High CO2-Capture Ability of a Porous Organic Polymer Bifunctionalized with Carboxy and Triazole Groups. Chem. Eur. J..

[3] Yuan W., Zhang X., Zhao J., Li Q., Ao C., Xia T., Zhang W., Lu C. (2017). Ultra-lightweight and highly porous carbon aerogels from bamboo pulp fibers as an effective sorbent for water treatment. Results Phys..

[4] Xu P., Drewes J.E., Heil D., Wang G. (2008). Treatment of brackish produced water using carbon aerogel-based capacitive deionization technology. Water Res..

[5] Thapliyal P.C., Singh K. (2014). Aerogels as Promising Thermal Insulating Materials: An Overview. J. Mater..

[6] Feng J., Zhang C., Feng J. (2012). Carbon fiber reinforced carbon aerogel composites for thermal insulation prepared by soft reinforcement. Mater. Lett..

[7] Li J., Wang X., Huang Q., Gamboa S., Sebastian P.J. (2006). Studies on preparation and performances of carbon aerogel electrodes for the application of supercapacitor. J. Power Sources.

[8] Al-Muhtaseb S.A., Ritter J.A. (2003). Preparation and Properties of Resorcinol–Formaldehyde Organic and Carbon Gels. Adv. Mater..

[9] Taylor S.J., Haw M.D., Sefcik J., Fletcher A.J. (2014). Gelation Mechanism of Resorcinol-Formaldehyde Gels Investigated by Dynamic Light Scattering. Langmuir.

[10] Shen J., Hou J., Guo Y., Xue H., Wu G., Zhou B. (2005). Microstructure Control of RF and Carbon Aerogels Prepared by Sol-Gel Process. J. Sol-Gel Sci. Technol..

[11] Awadallah-F A., Elkhatat A., Al-Muhtaseb S. (2011). Impact of synthesis conditions on meso- and macropore structures of resorcinol–formaldehyde xerogels. J. Mater. Sci..

[12] Prostredny M., Fletcher A., Mulheran P. (2019). Modelling the formation of porous organic gels – how structural properties depend on growth conditions. RSC Adv..

[13] Díez Orrite S., Stoll S., Schurtenberger P. (2005). Off-lattice Monte Carlo simulations of irreversible and reversible aggregation processes. Soft Matter.

[14] Lattuada M., Wu H., Morbidelli M. (2003). A simple model for the structure of fractal aggregates. J. Colloid Interface Sci..

[15] Rottereau M., Gimel J.C., Nicolai T., Durand D. (2004). Monte Carlo simulation of particle aggregation and gelation: I. Growth, structure and size distribution of the clusters. Eur. Phys. J. E.

[16] Jungblut S., Joswig J.-O., Eychmüller A. (2019). Diffusion-Limited Cluster Aggregation: Impact of Rotational Diffusion. J. Phys. Chem. C.

[17] Jungblut S., Joswig J.-O., Eychmüller A. (2019). Diffusion- and reaction-limited cluster aggregation revisited. Phys. Chem. Chem. Phys..

[18] Meakin P. (1983). Formation of Fractal Clusters and Networks by Irreversible Diffusion-Limited Aggregation. Phys. Rev. Lett..

[19] Kolb M., Botet R., Jullien R. (1983). Scaling of Kinetically Growing Clusters. Phys. Rev. Lett..

[20] Gavalda S., Kaneko K., Thomson K.T., Gubbins K.E. (2001). Molecular modeling of carbon aerogels. Colloids Surf. A.

[21] Gavalda S., Gubbins K.E., Hanzawa Y., Kaneko K., Thomson K.T. (2002). Nitrogen Adsorption in Carbon Aerogels:  A Molecular Simulation Study. Langmuir.

[22] Tamon H., Ishizaka H. (2000). Influence of Gelation Temperature and Catalysts on the Mesoporous Structure of Resorcinol–Formaldehyde Aerogels. J. Colloid Interface Sci..

[23] Horikawa T., Hayashi J.I., Muroyama K. (2004). Controllability of pore characteristics of resorcinol–formaldehyde carbon aerogel. Carbon.

[24] Pekala R.W. (1989). Organic aerogels from the polycondensation of resorcinol with formaldehyde. J. Mater. Sci..

[25] Pekala R.W., Schaefer D.W. (1993). Structure of organic aerogels. 1. Morphology and scaling. Macromolecules.

[26] Tamon H., Ishizaka H. (1998). SAXS Study on Gelation Process in Preparation of Resorcinol–Formaldehyde Aerogel. J. Colloid Interface Sci..

[27] Berthon S., Barbieri O., Ehrburger-Dolle F., Geissler E., Achard P., Bley F., Hecht A.-M., Livet F., Pajonk G.M., Pinto N. (2001). DLS and SAXS investigations of organic gels and aerogels. J. Non-Cryst. Solids.

[28] Alshrah M., Mark L.H., Zhao C., Naguib H.E., Park C.B. (2018). Nanostructure to thermal property relationship of resorcinol formaldehyde aerogels using the fractal technique. Nanoscale.

[29] Data for: “Modelling Organic Gel Growth in Three-Dimensions: Textural and Fractal Properties of Resorcinol-Formaldehyde Gels”. https://pureportal.strath.ac.uk/en/datasets/data-for-modelling-organic-gel-growth-in-three-dimensions-textura.

[30] Prostredný M., Abduljalil M.G.M., Mulheran P.A., Fletcher A.J. (2018). Process Variable Optimization in the Manufacture of Resorcinol⁻Formaldehyde Gel Materials. Gels.

[31] Bowler N.E., Ball R.C. (2005). Off-lattice noise reduced diffusion-limited aggregation in three dimensions. Phys. Rev. E.

[32] Tange O. (2011). GNU Parallel—The Command-Line Power Tool. USENIX Mag..

[33] Lin C., Ritter J.A. (1997). Effect of synthesis pH on the structure of carbon xerogels. Carbon.

[34] Grassberger P., Procaccia I. (1983). Characterization of Strange Attractors. Phys. Rev. Lett..

[35] Addison P.S. (1997). Fractals and Chaos: An Illustrated Course.

