# Supplementary material for: Modelling Organic Gel Growth in Three Dimensions: Textural and Fractal Properties of Resorcinol–Formaldehyde Gels
Source: Gels. 2020 Aug 5;6(3):23. doi: 10.3390/gels6030023 (PMC7559592; doi:10.3390/gels6030023)
Supplement: Supplementary file 1 [file gels-06-00023-s001.pdf]

gels

Supplementary Materials

Correlation Dimension Calculations

Figures S1 and S2 are supporting data for the calculation of the correlation dimension (D_C), which is determined from the gradient of the straight line section within the log-log plot of correlation sum (C_r) versus the spanning radius (r). This is shown for varying activated monomer percentage (C_C) in Figure S1 and varying solids content (S_C) in Figure S2. The initial scattering of data points (typically below $\log r$ values of around 1) arise due to the discrete nature of the lattice, whilst the plateau observed above $\log r$ values of around 2 is due to large scale effects of the periodic boundary conditions. The straight line section between these two limits is known as the scaling region, and this is the section used to calculate D_C.

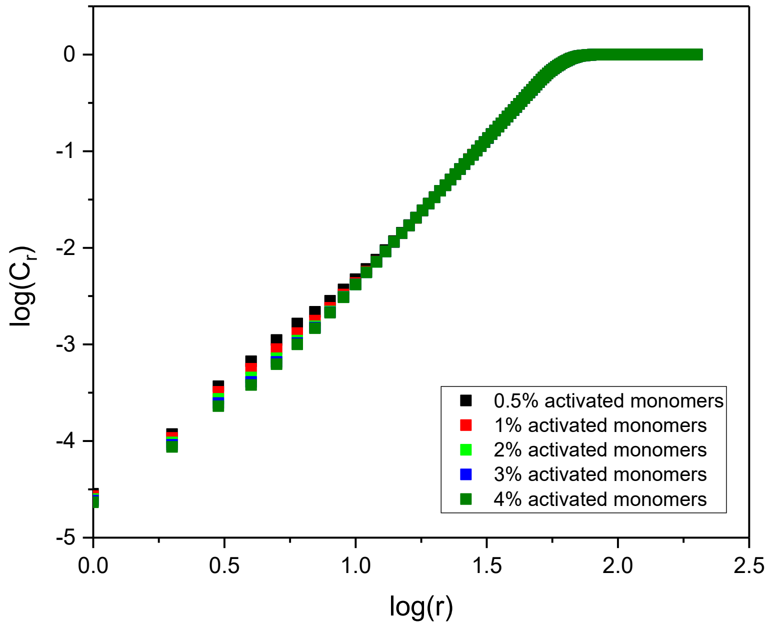

Figure S1. Log-log scale plot of the correlation sum (C_r) versus the spanning radius (r) at 20% solids content, with varying activated monomer percentages.

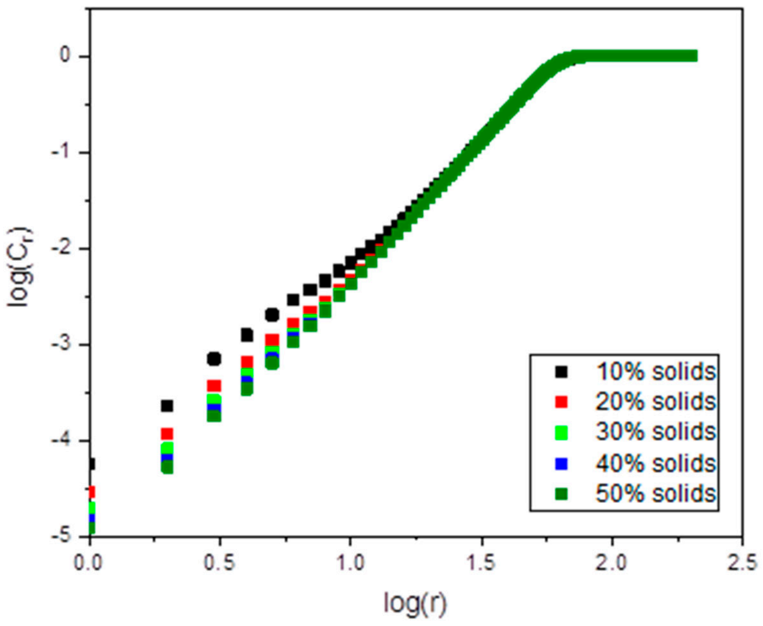

Figure S2. Log-log scale plot of the correlation sum (C_r) versus the spanning radius (r) at 0.5% activated monomers, varying solids content percentages.

Hurst Exponent Calculations

Figures S3 and S4 are supporting data for the calculation of the Hurst exponent (H), which is determined from the gradient of the straight line section within the log-log plot of the average displacement (ΔB_{avg}) versus the time window (T_s). This is shown for varying activated monomer percentage (C_C) in Figure S3 and varying solids content (S_C) in Figure S4, both of which are for a diffusing particle of 3 sites in size.

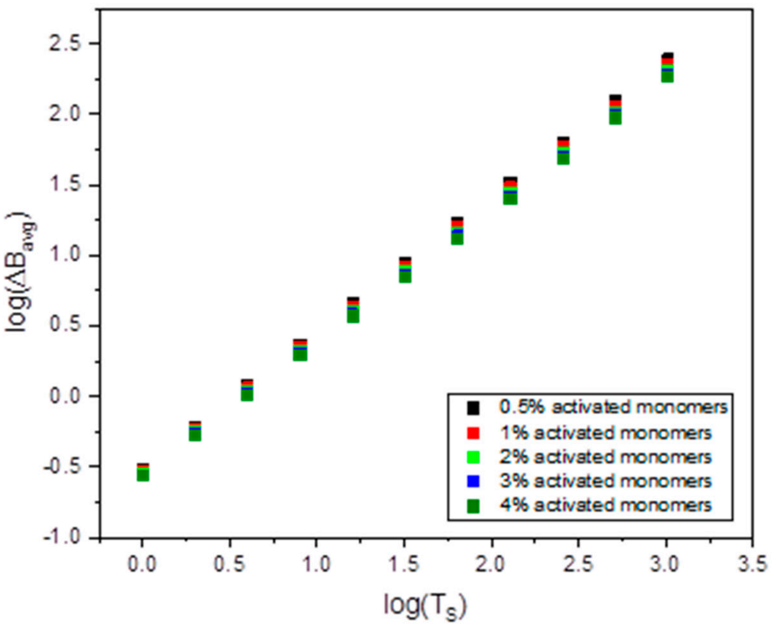

Figure S3. Log-log scale plot of the average displacement (ΔB_{avg}) versus the time window (T_s) at 20% solids content, with varying activated monomer percentages.

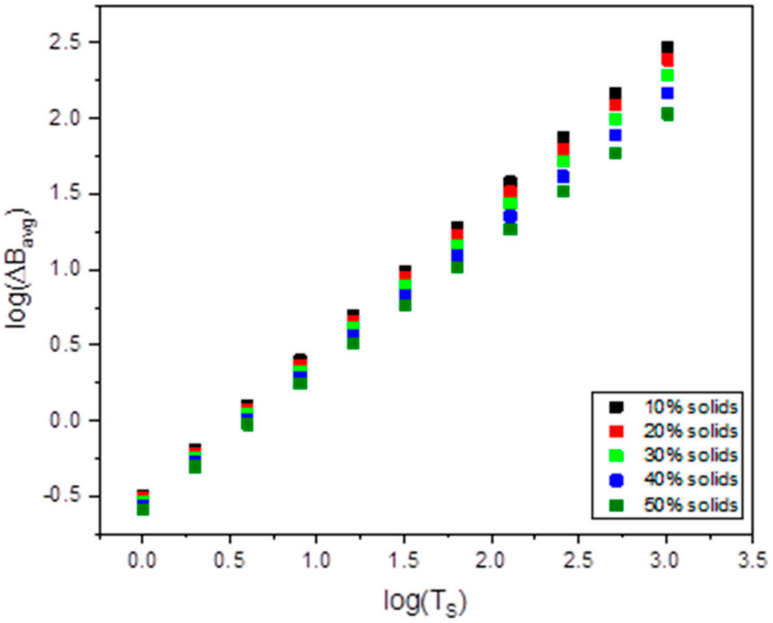

Figure S4. Log-log scale plot of the average displacement (ΔB_{avg}) versus the time window (T_s) at 0.5% activated monomers, varying solids content percentages.
